# Taking Mental Health into Schools: The work of Headucate Student Society

**DOI:** 10.1192/j.eurpsy.2022.190

**Published:** 2022-09-01

**Authors:** N. Henderson, V. Selwyn, R. Howard, I. Bartolome

**Affiliations:** University of East Anglia, Norwich Medical School, Norwich, United Kingdom

## Abstract

Headucate: University of East Anglia (UEA) is a student-led organisation aiming to raise awareness around Mental Health through their workshops and events. Events in the last year have included a 24-hour live stream TV fundraiser, UEA’s first Psychiatry and Mental Health conference, a mental health themed stand-up show, mental health assistance workshops, a suicide prevention evening and a variety of panels. Headucate has also collaborated with Beat, the UK’s leading eating disorder charity, to run training aimed at Norwich Medical School students, other healthcare students and Professionals. Headucate provides opportunities for their members, including research, education within the medical community, organising group projects, hosting poster competitions and by collaborating with likeminded initiatives. Workshops were designed to include common mental health conditions; wellbeing; advice regarding looking after yourself and others; ways to get for support and the impact of COVID-19 on our mental health. They are arranged and delivered by our members to school children and university students; they have been adapted to an online platform, which allowed us to reach a wider audience. Outcome measures demonstrating the impact of Headucate’s work were analysed via verbal, written and rated feedback, obtained from participants of workshops and events. Headucate workshops can help to improve mental health awareness and education in schools; however, it is only a local initiative. To educate schools outside of Norfolk, Headucate’s repeatable model can be used to create new branches of organisations that can spread nationwide; so mental health education in schools can be promoted across the UK.

**Disclosure:**

No significant relationships.

